# Influencing factors associated with high myopia in Chinese college students

**DOI:** 10.3389/fmed.2023.1146291

**Published:** 2023-06-23

**Authors:** Weiran Zhang, Xiaoyun Hou, Chang Li, Sennan Wang, Nianen Liu, Yan Zhang, Zhiqing Li

**Affiliations:** Tianjin Key Laboratory of Retinal Functions and Diseases, Tianjin Branch of National Clinical Research Center for Ocular Disease, Eye Institute and School of Optometry, Tianjin Medical University Eye Hospital, Tianjin, China

**Keywords:** myopia, vessel density, retinal thickness, habits, lifestyles, influencing factor

## Abstract

**Background:**

High myopia (HM) may elicit irreversible pathological changes in the fundus and severely impair visual quality, thereby becoming a major public health issue in China. However, the influencing factors associated with HM remain unknown in Chinese college students, whose visual quality is crucial to country development.

**Methods:**

This is a cross-sectional observational study. Two thousand three hundred and fifteen undergraduate and graduate students were initially recruited from various majors in 3 universities in Tianjin, China. Under the principle of voluntary participation and informed consent, simple random sampling was conducted in the recruited subjects while maintaining balanced number of subjects from each major. After screening with inclusion and exclusion criteria, 96 undergraduate and graduate students (186 eyes) were finally included and divided into non-HM and HM groups. The eyes of subjects were examined by optical coherence tomography angiography (OCTA) for vessel density and structure thickness at the macula and optic disc, and the subjects were surveyed by an itemized questionnaire on lifestyles and study habits.

**Results:**

The OCTA and questionnaire results revealed 10 factors, including hemodynamic and anatomic parameters and lifestyle metrics, with statistical significance between the non-HM and HM groups. Receiver operating characteristic curve analysis showed that vessel density of the inner retina at the macula, vessel density of the radial peripapillary capillary at the optic disc, smartphone usage time, continuous near work time, and sleeping after midnight had superior values of area under the curve (AUC > 0.700). Therefore, these 5 factors were selected for univariant and multivariant logistic regression analyses. A prediction model comprising the 5 influencing factors had an AUC of 0.940 and 95% CI of 0.908–0.972.

**Conclusion:**

This study for the first time identified the vessel density of the inner retina at the macula, the vessel density of the radial peripapillary capillary at the optic disc, smartphone usage time, continuous near work time, and sleeping after midnight as influencing factors associated with HM in Chinese college students. A prediction model comprising the 5 influencing factors was proposed for calculating likelihood of a Chinese college student developing HM, based on which lifestyle improvement and medical intervention might be recommended.

## 1. Introduction

Myopia is an epidemic around the globe. According to epidemiological reports, 34% of the world’s population was myopic in 2020, and this number will increase to 49.8% by 2050 (1). The situation is more severe in China, where the incidence of myopia even reaches 90% among high school students (2). Furthermore, the prevalence of high myopia (HM, refractive error greater than −6.00 diopters) in this country is estimated to double by 2050 (1). HM may elicit irreversible pathological changes in the fundus, such as posterior staphyloma, choroidal neovascularization, and chorioretinal atrophy (3), which in turn will cause uncorrectable impairments in visual quality. If no effective measures are taken, a significant proportion of the population will suffer from HM and the consequent decline in visual quality. Therefore, it is of urgent need to understand HM-associated influencing factors, based on which effective preventative and control measures can be implemented to delay the progression and attenuate the exacerbation of HM.

The influencing factors for HM remain incompletely understood. Both genetic and environmental factors play crucial roles in the occurrence and progression of HM (4). Genetic variations, such as single nucleotide polymorphism and insertion and deletion of genes, impart the individual’s susceptibility to HM, yet genetic factors alone cannot explain the surged prevalence of HM across the wide ethnic groups of China within a relatively short time period (5). Therefore, the environmental changes brought about by contemporary lifestyles, including but not limited to coronavirus lockdown, reduced outdoor activities, long hours of near work, heavy usage of electronic terminals, and educational and professional stress, attract attention from researchers and the administration (4).

Most studies on influencing factors associated with HM are focusing on children and adolescents (6–8). However, a retrospective cohort study conducted by Du et al. (9) have revealed continuous axial length (AL) elongation in the adults with HM (9). Moreover, a longitudinal study of a large Chinese cohort also demonstrated continuous myopia progression and AL elongation in the HM subjects at all ages, particularly in the young participants who have passed their adolescent stage (10). These studies implicate the possibility of continuous HM progression throughout life and highlight the importance of studying the influencing factors of HM in young adults. In China, college students, such as undergraduates and graduates in universities, are the key group of population among the young adults that should be protected from progression and exacerbation of HM. Because their eyes have been heavily used during primary and high school education (11), and they are still undergoing rapid development and progression of HM due to the abovementioned changes in lifestyles (12). On the other hand, impaired visual function is independently associated with an increased risk of work incompetence, unemployment, and being in a lower occupational category and having a lower household income (13). Therefore, the HM-induced severe visual impairments in Chinese college students may generate significant socioeconomic burdens to both families and the country, thereby prioritizing the study of the influencing factors associated with HM in this population.

Basic research has shown that the reduced choroidal blood perfusion is both sufficient and necessary to myopigenesis in guinea pigs (14, 15). Moreover, longitudinal clinical studies found that improving choroidal blood flow and thickness, through topic application of atropine (16) or low-level red-light illumination (17), could inhibit myopia progression, suggesting a pathogenic role of reduced choroidal circulation in myopia occurrence and development. Yet the changes in choroidal blood circulation are difficult to detect due to its deep anatomic location and fast hemodynamics. Interestingly, 2 recent studies using optical coherence tomography angiography (OCTA) identified reductions in vessel density and structural thickness in the retina in parallel to those in the choroid in the subjects with nonpathologic myopia (18, 19). Another study entailing OCTA demonstrated improvements in retinal blood flow and thickness induced by 3-month wear of orthokeratology lens and concomitant effective myopia control (20). These recent studies implicate that the vascular and anatomical changes detected by OCTA in the retina could at least reflect the choroidal changes that are pathogenic to myopia initiation and progression.

Therefore, this study sought to identify, for the first time, the influencing factors associated with HM in Chinese college students by examining the hemodynamic and anatomic metrics in the retina using OCTA and by surveying the parameters of lifestyle using a customized questionnaire. The results were then thoroughly analyzed by statistical methods such as receiver operating characteristic curve (ROC) analysis and univariant and multivariant logistic regression analyses.

## 2. Materials and methods

This is a cross-sectional observational study conducted from 20th December 2021 to 15th March 2022, during which undergraduate and graduate students in universities in Tianjin, China, were recruited. The study adhered to the tenets of the Declaration of Helsinki and was approved by the ethical review committee of Tianjin Medical University Eye Hospital (2022KY(L)-13). Written informed consent was acquired from all the subjects prior to recruitment to this study. The study was registered at the Chinese Clinical Trial Registry (ChiCTR2100054423).

### 2.1. Subjects

Three universities, including Nankai University, Tianjin University, and Tianjin Medical University, were selected for the study. The 3 universities, with enrollment of more than 100,000 undergraduate and graduate students, represent the institutions of higher education in science, engineering, and medical profession, respectively, in Tianjin, China. Initially, 2,315 undergraduate and graduate students were recruited from various majors, such as electronic information, chemistry, computer science, precision instruments, biomedical engineering, optometry, and clinical medicine. Subsequently, under the principle of voluntary participation and informed consent, simple random sampling was performed in the initially recruited subjects while ensuring balanced number of subjects selected from each major. After this step, 105 subjects (210 eyes) were included for the study.

The following formula was used for sample size calculation.


$$n=\frac{Z_{1-\frac{\alpha }{2}}^{2}\times p\;\left(1-p\right)}{d^{2}}$$

Based on the literature report, the prevalence of HM (*p*) is 15% in the general population. The α value was set as 0.05, and d was considered as 0.20 × *p*.

### 2.2. Inclusion and exclusion criteria

Inclusion criteria: (1) college students at the age of 18 to 28 years; (2) best corrected visual acuity (BCVA) ≥ 0.8; (3) intraocular pressure ≤ 21 mmHg (1 mmHg = 0.133 kPa); (4) spherical equivalent refraction (SER) ≤ −0.50 D. Exclusion criteria: (1) BCVA < 0.8; (2) history of systemic diseases, such as hypertension and diabetes; (3) history of intraocular or refractive surgery; (4) intraocular pressure > 21 mmHg or normal tension glaucoma; (5) unable to receive examinations due to poor fixation or opacity of refractive media; (6) SER > −0.50 D. According to these criteria, 24 eyes were excluded, and the remaining 186 eyes were included in the study.

### 2.3. Stratification of the subjects

The subjects in this study were divided into 2 groups according to the non-cycloplegic SER: non-HM group (− 6.00 D < SER ≤ −0.50 D) and HM group (SER ≤ −6.00 D), based on the literature report (21). If both eyes of the subject met the criteria, both eyes were included in the study. If one eye of the subject met the inclusion criteria, that eye was included, the eye that did not was excluded.

### 2.4. Ophthalmic examinations

All participants underwent ophthalmic examinations, including non-cycloplegic refractive error assessment (SW-800; Suoer, Tianjin, China) and SER conversion, BCVA assessment, intraocular pressure measurement (CT-80, Topcon, Tokyo, Japan), color fundus photography (CR-2; Canon, Tokyo, Japan), and measurement of AL (SW-9000; Suoer, Tianjin, China), as previously described (19).

### 2.5. Optical coherence tomography angiography

#### 2.5.1. Parameters of optical coherence tomography angiography

OCTA was performed using a spectral domain system RTVue XR Avanti (Optovue, Fremont, CA, United States) device that uses the algorithm of split-spectrum amplitude-decorrelation angiography (SSADA). The algorithm detects and analyzes the motion contrast of the red blood cells in the vessels through consecutive scans of a certain fundus location. The OCTA device was equipped with a light source with wavelength of 840 nm and bandwidth of 50 nm. The scan speed of the device is 70,000 axial scans/s, with horizontal and axial resolutions of 20 and 5 μm, respectively. Fundus scan protocols with a scan area of 6 × 6 mm^2^ at the macula and a scan area of 4.5 × 4.5 mm^2^ at the optic disc were selected for image acquisition. Every OCTA image was obtained by performing two repeated 304 B-scans, each of which was achieved by conducting 304 A-scans. Motion correction and minimization of motion artifacts were performed by software, and automatic segmentation was employed to distinguish different layers of the retina. The scanned images with a quality lower than 6 (up to 10) were deleted, and rescanning was performed.

#### 2.5.2. Definition of the area around the macula

**Figure 1:**
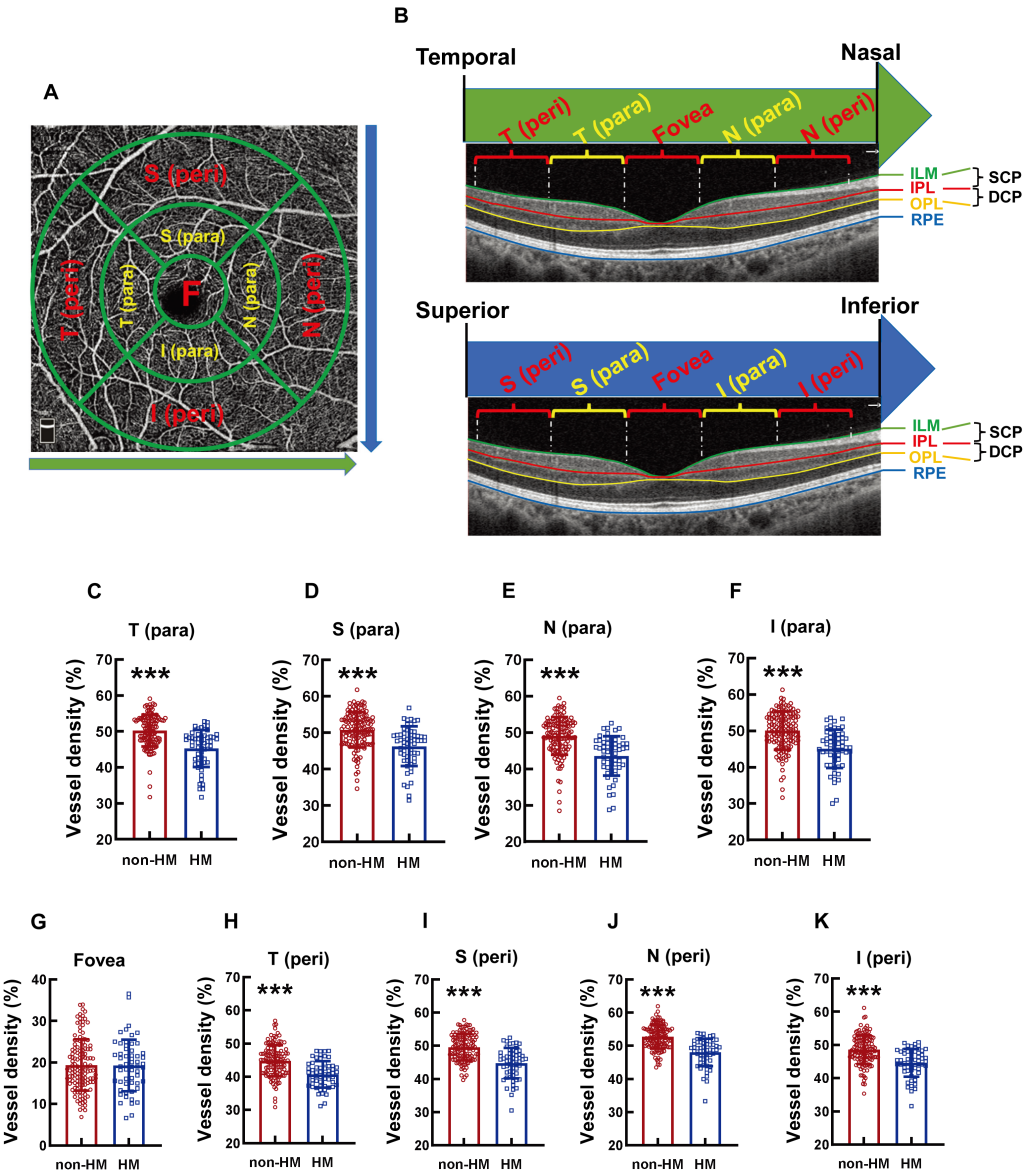
Comparison of the vessel densities of different parts of the macula at the SCP level between the non-HM and HM groups. The compartmentalization of the macula is shown at an enface image of the OCTA **(A)**. The macular compartments, the SCP and DCP levels were illustrated in a cross-sectional image automatically acquired by the built-in software **(B)**. The green and red lines indicate the upper (ILM) and lower (IPL) boundaries of the SCP, respectively; The red and yellow lines indicate the upper (IPL) and lower (OPL) boundaries of the DCP, respectively. The green arrow indicates the direction from the temporal to the nasal. The blue arrow indicates the direction from the superior to the inferior. The vessel densities at the T para **(C)**, S para **(D)**, N para **(E)**, I para **(F)**, fovea **(G)**, T peri **(H)**, S peri **(I)**, N peri **(J)**, and I peri **(K)** parts at the SCP level were compared between the non-HM and HM groups. ^***^p < 0.001. SCP, superficial capillary plexus; DCP, deep capillary plexus; T para, temporal parafovea; S para, superior parafovea; N para, nasal parafovea; I para, inferior parafovea; T peri, temporal perifovea; S peri, superior perifovea; N peri, nasal perifovea; and I peri, inferior perifovea; ILM, internal limiting membrane; IPL, inner plexiform layer; RPE, retinal pigmented epithelium; non-HM, nonhigh myopia; HM, high myopia.

According to the partition criteria described in the Early Treatment of Diabetic Retinopathy Study (22), the area around the macula was defined by 3 cocentric circles with diameters of 1, 3, and 5 mm. The circle with a diameter of 1 mm was defined as the fovea. The area between the 1 mm-diameter circle and the 3 mm-diameter circle was divided into 4 parts, designated as temporal parafovea (T para), superior parafovea (S para), nasal parafovea (N para), and inferior parafovea (I para) in a clockwise manner (Figure 1A). The area between the 3 mm-diameter circle and the 5 mm-diameter circle was also divided into 4 parts: temporal perifovea (T peri), superior perifovea (S peri), nasal perifovea (N peri), and inferior perifovea (I peri; Figure 1A).

#### 2.5.3. Definition of the area around the optic disc

**Figure 2:**
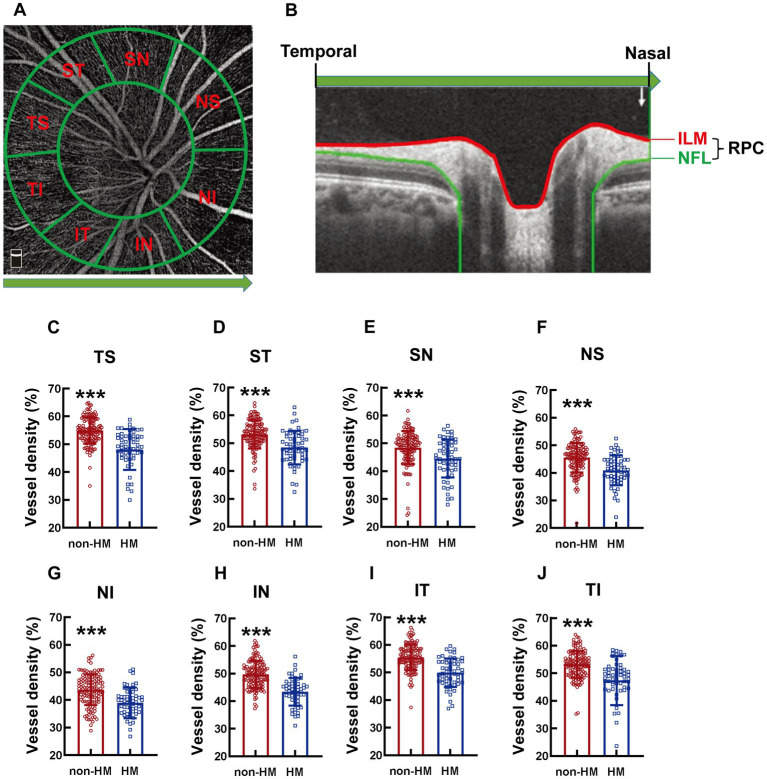
Comparison of the RPC vessel densities of different parts of the optic disc between the non-HM and HM groups. The compartmentalization of the optic disc is illustrated in an en face image of OCTA **(A)**. A cross-sectional image was automatically obtained by the built-in software **(B)**. The red and blue lines indicate the upper (ILM) and lower (NFL) boundaries of the RPC. The green arrow represents the direction from the temporal to the nasal. The RPC vessel densities at the TS **(C)**, ST **(D)**, SN **(E)**, NS **(F)**, NI **(G)**, IN **(H)**, IT **(I)**, and TI **(J)** parts of the optic disc were compared between the non-HM and HM groups. ^***^p < 0.001. NS, nasal superior; NI, nasal inferior; IN, inferior nasal; IT, inferior temporal; TI, temporal inferior; TS, temporal superior; ST, superior temporal; SN, superior nasal; ILM, internal limiting membrane; NFL, nerve fiber layer; non-HM, nonhigh myopia; HM, high myopia.

The area around the optic disc was defined by 2 cocentric circles with diameters of 2 and 4 mm and with the center at the optic disc. The area between the 2 circles was divided into 8 parts, which were termed clockwise as nasal superior (NS), nasal inferior (NI), inferior nasal (IN), inferior temporal (IT), temporal inferior (TI), temporal superior (TS), superior temporal (ST), and superior nasal (SN; Figure 2A).

#### 2.5.4. The vessel density and structural thickness at the macular and the optic disc

The superficial capillary plexus (SCP) was defined as the vasculature between the inner limiting membrane (ILM) and 9 μm above the inner plexiform layer (IPL), whereas the deep capillary plexus (DCP) referred to the vasculature between 9 μm above the IPL and 9 μm below the outer plexiform layer (OPL). The vessel densities at the abovementioned 9 parts (T para, S para, N para, I para, T peri, S peri, N peri, I peri, and fovea) of the macula can be measured by OVTA at both the SCP and DCP levels (Figure 1B). The ensemble vessel densities at the macula can also be measured at these 2 levels, the mean value of which was used to represent the vessel density of inner retina at the macula. The thicknesses at the abovementioned 9 parts of the macula as well as that at the ensemble macula all refer to the thickness between the ILM and the retinal pigmented epithelium (RPE). The radial peripapillary capillary (RPC) was defined as the vasculature between the ILM and the nerve fiber layer (NFL). The vessel density at the optic disc is the vessel density of the RPC, either at the parts or at the whole optic disc. The structural thickness at the optic disc measures the thickness of the RPC, either at the parts or at the whole optic disc.

### 2.6. Questionnaire

A questionnaire survey was conducted for each included subject. The survey focuses on the parameters of study habits and lifestyles that may affect the occurrence and progression of HM (Supplementary Figure 1). The questionnaire includes the following questions: (1) Profession; (2) Sex; (3) Grade; (4) How long have you been myopic; (5) How many hours do you spend on smartphone every day; (6) How many hours do you use computer every day; (7) How many hours do you read books every day; (8) How many hours is the longest continuous use of your eyes every day; (9) How much time do you spend on outdoor sports every day; (10) How many hours do you sleep every day; and (11) When do you go to bed every day (Supplementary Figure 1). The replies to each question were recorded and quantified by an assistant unaware of the grouping. Values were assigned to the replies of the questions involving categorical variables: education background (undergraduate = 0, graduate = 1); major (medical profession = 0, nonmedical profession = 1); outdoor sport time every day (<1 h = 0, ≥ 1 h = 1); history of myopia (<5 years = 0, ≥5 years = 1); the time to fall asleep (before 12 a.m. = 0, after 12 a.m. = 1). The replies to the questions involving continuous variables were analyzed using the original values.

### 2.7. Statistics

Statistical Program for Social Sciences 25.0 (IBM SPSS Inc., New York, NY, United States) was used for statistical analysis. The sex distribution in each group was expressed as the ratio of females to males, and the difference in sex distribution between the 2 groups was compared using Chi-square test. When the data of both eyes of one subject were used for analysis, Bartlett’s test of sphericity was performed to ensure that the two eyes were not correlated. The continuous data were examined by D’Agostino and Pearson omnibus normality tests, after which they were examined by Levene’s test to determine homogeneity of variance. The data with Gaussian distribution and homogeneity of variance were expressed as the mean ± SD, and the differences between the 2 groups were analyzed using the two-sided unpaired t test. The data with a non-Gaussian distribution and those with a Gaussian distribution but an uneven variance were expressed as the median (interquartile range, 25 to 75%), and the differences between the 2 groups were analyzed using the Mann–Whitney *U*-test. The categorical data were analyzed by Chi-square test or Fisher’s exact test. *p* < 0.05 was deemed statistically significant.

Univariate logistic regression and receiver operating characteristic curve (ROC) analyses were performed on the vessel density and structural thickness at the macula, the vessel density and thickness of RPC at the optic disc, and the parameters exhibiting significant differences between the 2 groups in the questionnaire survey. Then, the factors with the value of area under the curve (AUC) > 0.700 were selected as variables for multivariant logistic regression analysis, aiming to determine the influencing factors associated with HM in these Chinese college students.

## 3. Results

### 3.1. Demographic information

**Figure 3:**
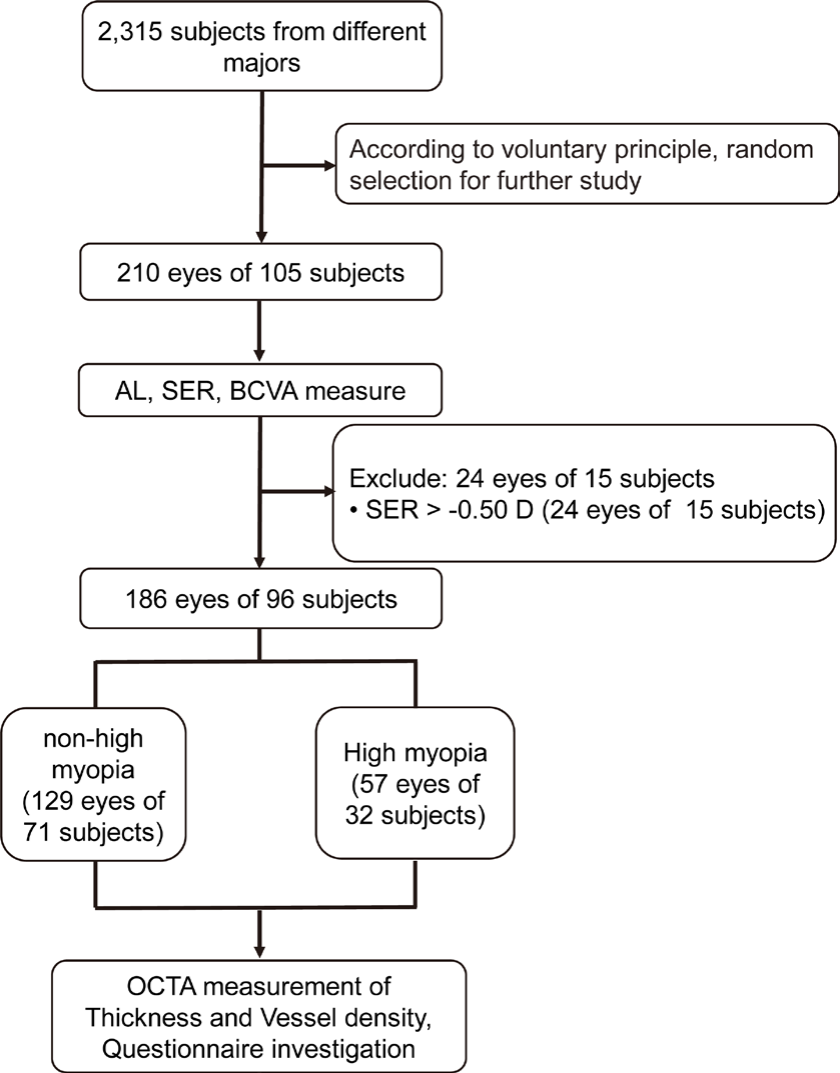
Flowchart for the current study. AL, axis length; SER, spherical equivalent refraction; BCVA, best-corrected visual acuity; OCTA, optical coherence tomography angiography.

Two hundred and ten eyes (105 subjects) were initially recruited. Twenty-four eyes (15 subjects) were excluded according to the exclusion criteria (Figure 3). The remaining 186 eyes (96 subjects) were divided into a non-HM group and a HM group according to the non-cycloplegic SER. The non-HM and HM groups had 129 (71 subjects) and 57 (32 subjects) eyes, respectively (Figure 3). Then, each included eye was examined by OCTA, and the included subjects were surveyed by a customized questionnaire on their study habits and lifestyles (Figure 3). There was no significant difference between the 2 groups in the age or sex distribution (*p* = 0.376 for age, *p* = 0.797 for sex distribution; Table 1). As expected, highly significant differences were found between the 2 groups in the AL and SER (*p* < 0.001 for AL, *p* < 0.001 for SER; Table 1).

**Table 1:** Information on the studied eyes.

	Non-HM	HM	
	(129 eyes)	(57 eyes)	*p*
Age (y), Mean (SD)	22.43 (2.71)	22.84 (2.96)	0.376^*^
Sex (female:male)	84:45	21:36	0.797^†^
AL (mm), mean (SD)	25.15 (0.97)	26.73 (1.02)	<0.001^*^
SER (D), mean (SD)	−3.20 (1.48)	−7.06 (1.20)	<0.001^*^

Non-HM, nonhigh myopia group; HM, high myopia group; SD, standard deviation; AL, axial length; SER, spherical equivalent refraction.

* Nonparametric test.

† Chi-squared test.

### 3.2. The vessel densities at different parts of the macula at the SCP level

The compartmentalization of the macula is illustrated in an en face image (Figure 1A). Both macular compartmentalization, the SCP and DCP levels are indicated in a cross-sectional image (Figure 1B). The vessel density at the T para part of the macula at the SCP level was significantly reduced in the college students with HM compared to those with non-HM, with the vessel density of the HM subjects being 90.11% of their non-HM counterparts (*p* < 0.001, non-HM vs. HM; Figure 1C). The vessel densities at other parts of the macula, including S para, N para, I para, T peri, S peri, N peri, and I peri parts, all displayed a similar trend between the 2 groups of subjects, with the vessel densities of the HM group being approximately 10% less than the non-HM group (all *p* < 0.001, non-HM vs. HM; Figures 1D–F, H). In contrast, the vessel density at the fovea, the central part of the macula, did not exhibit any significant difference between the 2 groups (*p* = 0.912, non-HM vs. HM; Figure 1G). These results suggest significant decreases in the vessel densities at various parts, except the fovea, of the macula in Chinese college students with HM in comparison to their non-HM counterparts.

### 3.3. The vessel density at different parts of the macula at the DCP level

**Figure 4:**
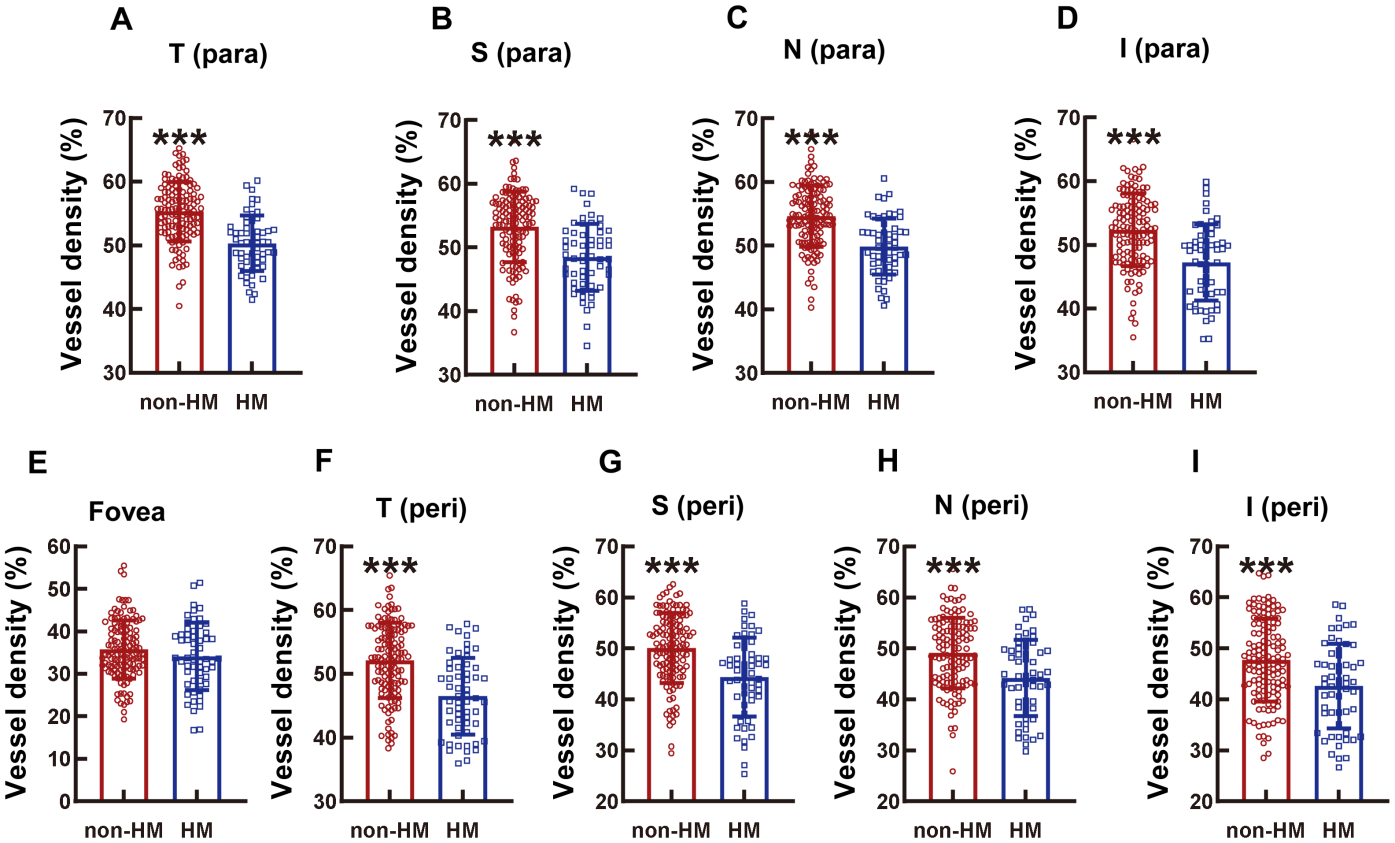
Comparison of the vessel densities of different parts of the macula at the DCP level between the non-HM and HM groups. The vessel densities at the T para **(A)**, S para **(B)**, N para **(C)**, I para **(D)**, fovea **(E)**, T peri **(F)**, S peri **(G)**, N peri **(H)**, and I peri **(I)** parts at the DCP level were compared between the non-HM and HM groups. ^***^p < 0.001. T para, temporal parafovea; S para, superior parafovea; N para, nasal parafovea; I para, inferior parafovea; T peri, temporal perifovea; S peri, superior perifovea; N peri, nasal perifovea; and I peri, inferior perifovea; non-HM, nonhigh myopia; HM, high myopia.

The absolute values of the vessel densities of the macular parts, such as all the para as well as the T and S peri parts, at the DCP level were higher than those of the corresponding parts at the SCP level (Figures 1, 4). The relative differences in the macular vessel densities at the DCP level between the 2 groups showed the trends similar to those at the SCP level, with the vessel densities of the non-HM subjects being approximately 10% higher than those of the HM subjects (all *p* < 0.001, non-HM vs. HM; Figures 4A–D, F). However, the vessel densities at the fovea at the DCP level were not significantly different between the non-HM and HM groups (*p* = 0.153, non-HM vs. HM; Figure 4E), which was consistent with the results at the SCP level (Figure 1G).

### 3.4. The thickness of the whole retina at different parts of the macula

**Figure 5:**
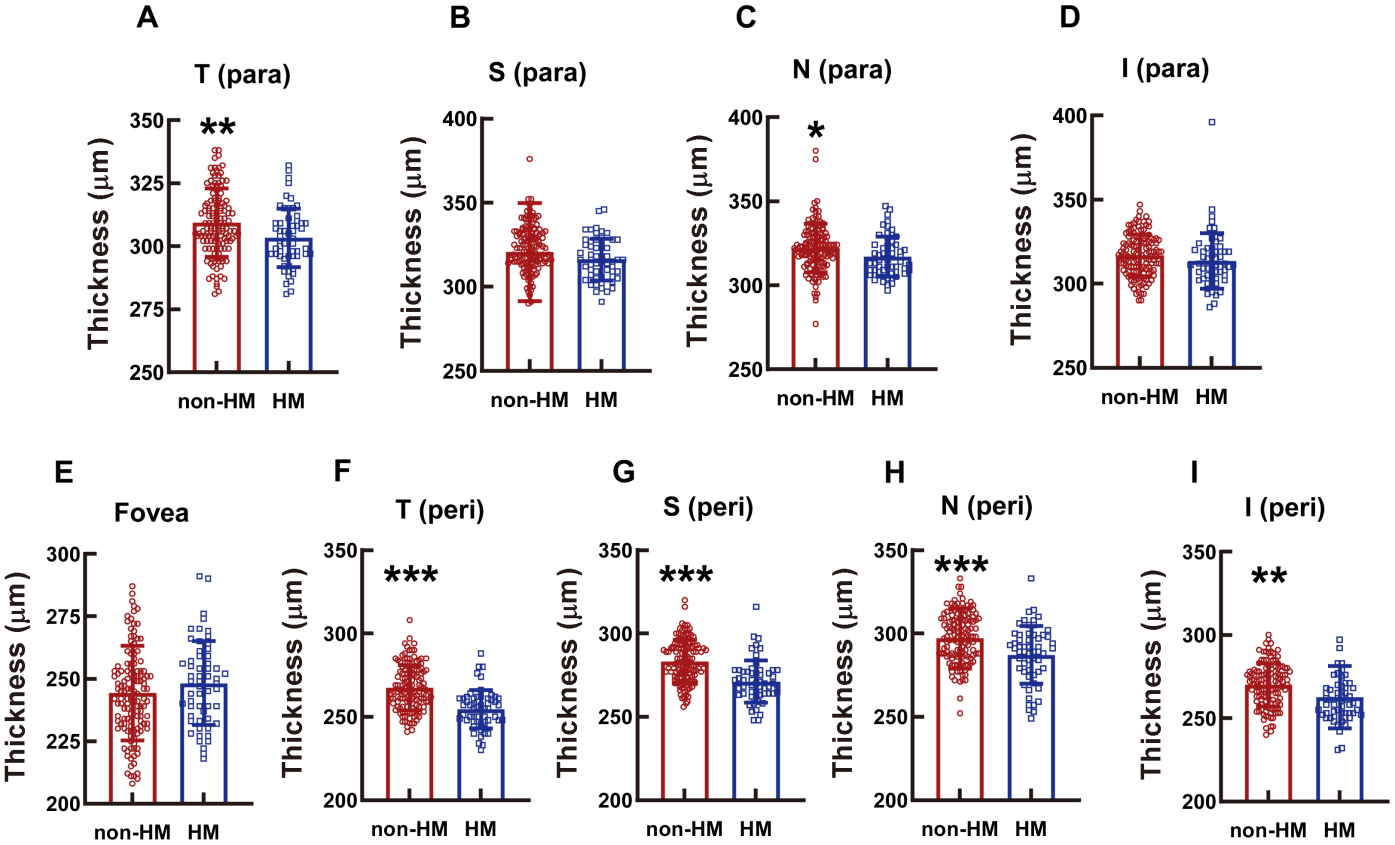
Comparison of the retinal thickness at the macula between the non-HM and HM groups. The retina thicknesses at the T para **(A)**, S para **(B)**, N para **(C)**, I para **(D)**, fovea **(E)**, T peri **(F)**, S peri **(G)**, N peri **(H)**, and I peri **(I)** parts were compared between the non-HM and HM groups. ^*^p < 0.05, ^**^p < 0.01, ^***^p < 0.001. T para, temporal parafovea; S para, superior parafovea; N para, nasal parafovea; I para, inferior parafovea; T peri, temporal perifovea; S peri, superior perifovea; N peri, nasal perifovea; and I peri, inferior perifovea; non-HM, nonhigh myopia; HM, high myopia.

The diminution in the whole retinal thickness of the HM subjects as opposed to the non-HM subjects was not obvious in the parafovea region; only the thicknesses at the T para and N para showed small but significant decrements (*p* < 0.01 for T para, *p* < 0.05 for N para, non-HM vs. HM; Figures 5A,C); no significant difference in the retina thickness was found at the other parafovea parts (*p* = 0.0277 for S para, *p* = 0.134 for I para, non-HM vs. HM; Figures 5B,D). In contrast, at all the perifoveal parts, the retina of the non-HM group was significantly thicker than that of the HM group (all *p* < 0.001 for T peri, S peri, and N peri, *p* < 0.01, for I peri, non-HM vs. HM; Figures 5F–I). Similar to the trend of the vessel density, the whole retina thicknesses of the 2 groups were not significantly different from each other at the fovea (*p* = 0.180, non-HM vs. HM; Figure 5E).

### 3.5. The vessel density at different parts of the optic disc

The compartmentalization of the optic disc is illustrated in an en face image (Figure 2A), and the RPC is indicated in a cross-sectional image (Figure 2B). The RPC vessel densities at various parts (TS, ST, SN, NS, NI, IN, IT, and TI) of the optic disc displayed a similar trend between the 2 groups across the board, with the vessel densities in the non-HM subjects being 8–12% greater than those in the HM subjects (all *p* < 0.001, non-HM vs. HM, Figures 2C–J).

### 3.6. The thickness at different parts of the optic disc

**Figure 6:**
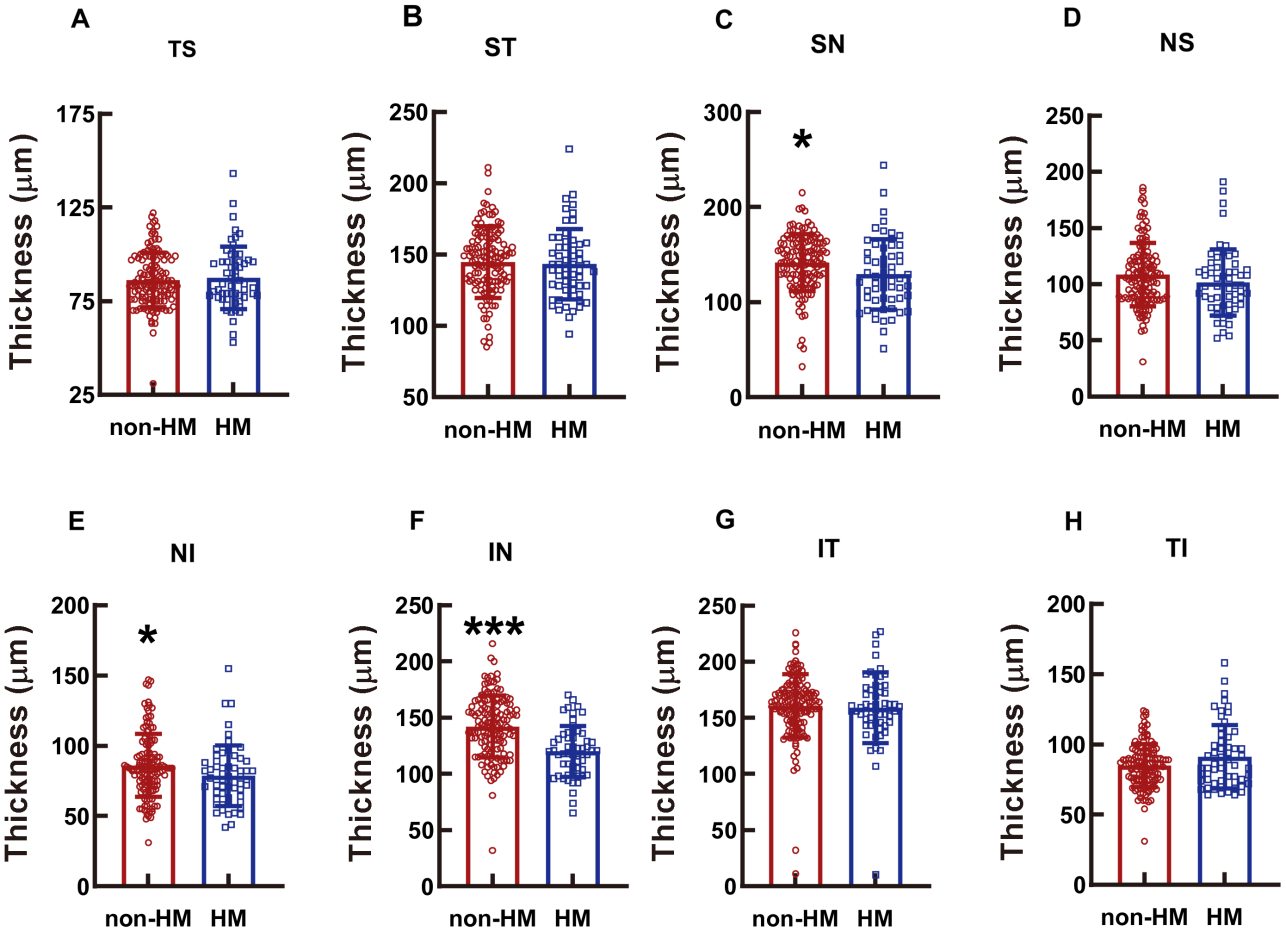
Comparison of the RPC thicknesses at different parts of the optic disc between the non-HM and HM groups. The RPC thicknesses at the TS **(A)**, ST **(B)**, SN **(C)**, NS **(D)**, NI **(E)**, IN **(F)**, IT **(G)**, and TI **(H)** parts of the optic disc were compared between the non-HM and HM groups. ^*^p < 0.05, ^***^p < 0.001. NS, nasal superior; NI, nasal inferior; IN, inferior nasal; IT, inferior temporal; TI, temporal inferior; TS, temporal superior; ST, superior temporal; SN, superior nasal; non-HM, nonhigh myopia; HM, high myopia.

The reduction in the RPC thickness in the HM subjects was significant at the SN, NI, and IN parts, with the thicknesses in the high myopes being 91.18%, 91.56%, and 84.42% of those in the nonhigh-myopic counterparts (*p* < 0.05 for SN, non-HM vs. HM, Figure 6C; *p* < 0.05 for NI, non-HM vs. HM, Figure 6E; *p* < 0.001 for IN, non-HM vs. HM, Figure 6F). The RPC thicknesses at other parts of the optic disc were not significantly different between the 2 study groups (*p* = 0.634 for TS, non-HM vs. HM, Figure 6A; *p* = 0.701 for ST, non-HM vs. HM, Figure 6B; *p* = 0.125 for NS, non-HM vs. HM, Figure 6D; *p* = 0.702 for IT, non-HM vs. HM, Figure 6G; *p* = 0.062 for TI, non-HM vs. HM, Figure 6H).

### 3.7. The ensemble vessel density and structural thickness at the macula and the optic disc

**Figure 7:**
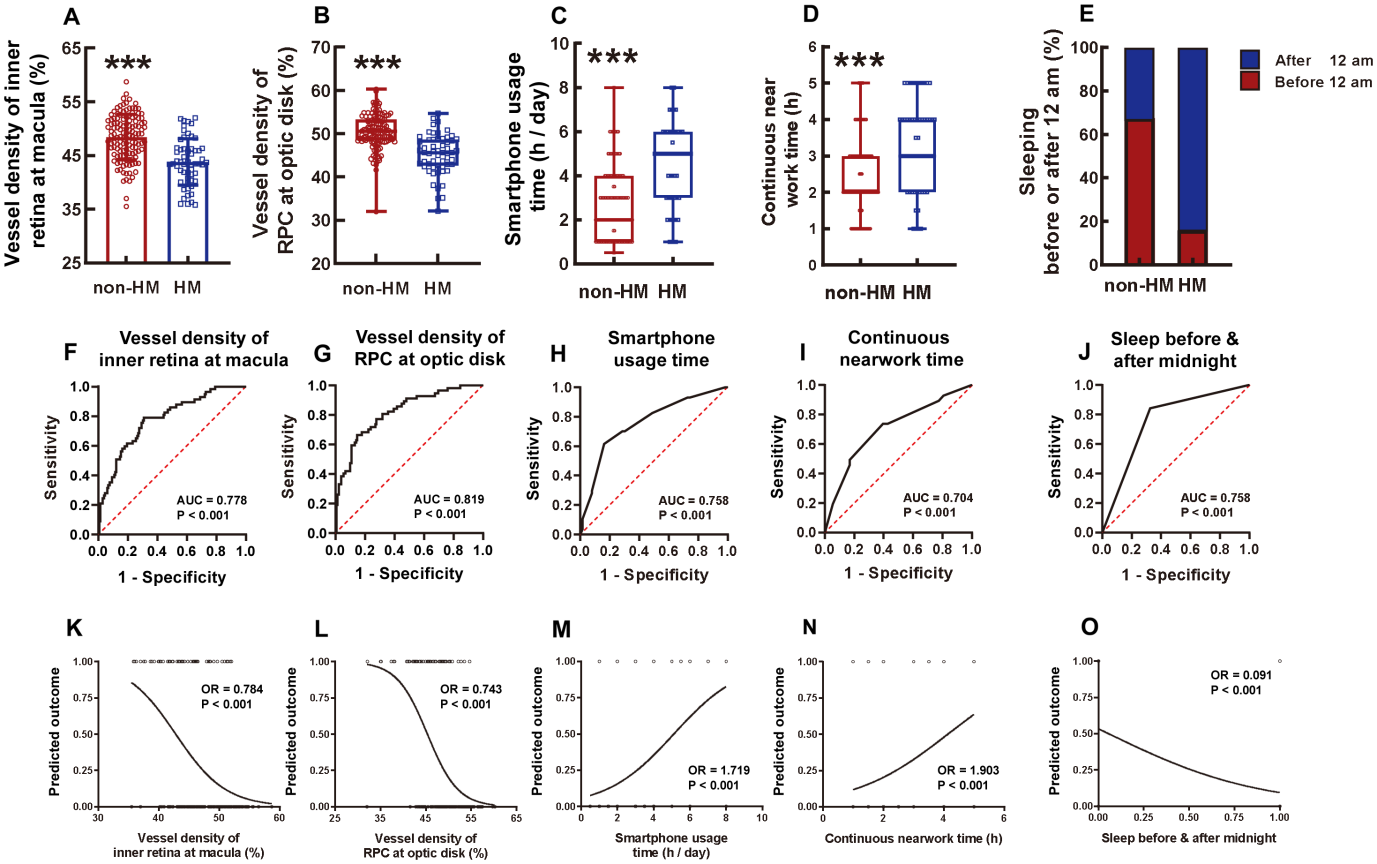
Comparison of 5 influencing factors between the non-HM and HM groups with a series of statistical analyses. The ensemble vessel densities of the inner retina segment ranging from the ILM to 9 μm below the OPL at the macula region were compared between the non-HM and HM groups **(A)**. The vessel densities of the RPC at the optic disc were compared between the non-HM and HM groups **(B)**. The smartphone usage time **(C)**, continuous near work time **(D)**, and sleeping before or after midnight **(E)** were compared between the non-HM and HM groups. The ROC curves of these 5 factors were shown in **(F–J)**. The univariate logistic analysis of these 5 factors were shown in **(K–O)**. ^***^p < 0.001. non-HM, nonhigh myopia; HM, high myopia; ROC, receiver operator characteristic; AUC, area under the curve; OR, odds ratio.

Consistent with the compartmentalized macula results, the ensemble macula vessel density of the inner retina was significantly higher in the non-HM group than in the HM group, with the former being 10% greater than the latter (*p* < 0.001, non-HM vs. HM, Figure 7A). The ensemble retina thickness at the macula in the non-HM group was also significantly increased compared to that in the HM group (*p* < 0.05, non-HM vs. HM, Supplementary Figure 1A), albeit the difference was not as great as the vessel density (Figure 7A; Supplementary Figure 1A).

Both the vessel density and thickness of RPC at the optic disc were significantly greater in the non-HM subjects than in the HM subjects (*p* < 0.001 for vessel density, non-HM vs. HM, Figure 7B; *p* < 0.05 for thickness, non-HM vs. HM, Supplementary Figure 1B). Taken together, the ensemble vessel density and structural thickness at both the macula and the optic disc reflected the fundamental differences between the 2 study groups and thereby may serve as representatives of the hemodynamic and anatomical factors impacting myopia progression in Chinese college students.

### 3.8. Questionnaire investigation

There were 5 continuous and 5 categorical parameters in the questionnaire. For the continuous parameters, the HM subjects had significantly greater smartphone usage time [HM 5.00 (3.00, 6.00) vs. non-HM 2.00 (1.00, 4.00)] and continuous near work time [HM 3.00 (2.00, 4.00) vs. non-HM 2.00 (2.00, 3.00)] (both *p* < 0.001, Figures 7C,D) but less sleeping time [HM 7.00 (7.00, 8.00) vs. non-HM 8.00 (7.00, 8.00)] (*p* < 0.001, Supplementary Figure 2A) on a daily basis than the non-HM subjects. No statistically significant difference was found in the everyday computer usage time [HM 4.00 (2.00, 8.00) vs. non-HM 5.00 (2.00, 6.00)] and the reading time [HM 3.00 (2.00, 4.00) vs. non-HM 2.00 (2.00, 3.00)] between the 2 groups (*p* = 0.221 for computer usage, *p* = 0.170 for book reading, non-HM vs. HM; Supplementary Figures 2B,C). For the categorical parameters, 67.44% of the nonhigh myopes slept before midnight, while merely 15.79% of the high myopes did so (*p* < 0.001, non-HM vs. HM, Figure 7E). Moreover, 53.49% of the non-HM subjects spent more than 1 h on outdoor sports every day, which, however, was accomplished by only 21.05% of the HM subjects (*p* < 0.001, non-HM vs. HM, Supplementary Figure 2D). Additionally, the majority (96.49%) of the HM group had a history of myopia of more than 5 years, whereas approximately half (58.14%) of the non-HM group had myopia longer than 5 years (*p* < 0.001, non-HM vs. HM, Supplementary Figure 2E). Grade (undergraduate or graduate) and major (medical or nonmedical profession) were not significantly different between the non-HM and HM groups in the Chinese college students (*p* = 0.548 for grade, *p* = 0.197 for major, non-HM vs. HM, Supplementary Figures 2F,G).

### 3.9. ROC analysis of each factor with statistical significance

The factors with statistical significance between the 2 study groups were selected and subjected to ROC analysis, which assessed the sensitivity and specificity of each factor to distinguish HM from non-HM. As for the hemodynamic and anatomical factors measured by OCTA, the vessel densities outperformed the structural thicknesses. The AUC of the ensemble vessel density of the inner retina at the macula was 0.778 (*p* < 0.001, Figure 7F), whereas that of the vessel density of RPC at the optic disc was 0.819 (*p* < 0.001, Figure 7G). Among the factors surveyed in the questionnaire, smartphone usage time, continuous near work time, and sleeping before or after midnight showed good performance, with AUCs of 0.758, 0.704, and 0.758, respectively (all *p* < 0.001, Figures 7H,I). These 5 factors were then included for further analysis of univariant logistic regression. Other factors, such as ensemble thickness of the retina at the macula (Supplementary Figure 3A), ensemble thickness of RPC at the optic disc (Supplementary Figure 3B), daily sleeping time (Supplementary Figure 3C), daily outdoor sport time (Supplementary Figure 3D), and history of myopia (Supplementary Figure 3E), were excluded from the logistic analysis due to their inferior distinguishing abilities (AUC < 0.700).

### 3.10. Univariate and multivariate logistic regression analysis

The results of univariate logistic regression analysis showed that the vessel density of the inner retina at the macula (Figure 7K), the vessel density of RPC at the optic disc (Figure 7L), the smartphone usage time (Figure 7M), the continuous near work time (Figure 7N), and whether sleeping before or after midnight (Figure 7O) were all able to significantly distinguish the non-HM group from the HM group (all *p* < 0.001).

**Figure 8:**
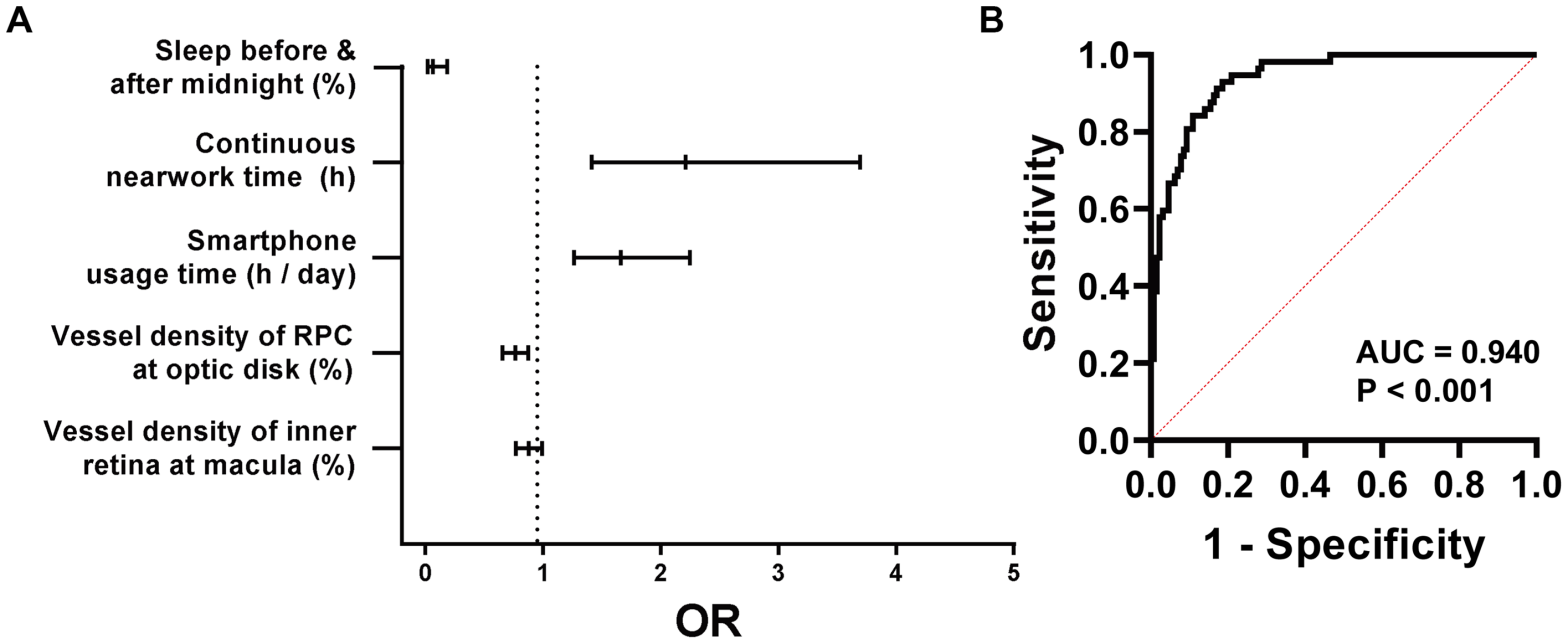
Multivariant logistic regression analysis of HM. The forest map of the ORs of these 5 factors is shown in **(A)**. A HM prediction model comprised of these 5 factors is proposed, the sensitivity and specificity of which are shown in **(B)**. HM, high myopia; AUC, area under the curve; ORs, odds ratios.

All these 5 factors were then selected for a multivariate regression analysis, which suggested that the vessel density of the inner retina at the macula (OR = 0.878, 95% CI: 0.769–0.992), the vessel density of RPC at the optic disc (OR = 0.762, 95% CI: 0.655–0.874), the smartphone usage time (OR = 1.658, 95% CI: 1.264–2.246), the continuous near work time (OR = 2.211, 95% CI: 1.411–3.694), and whether sleeping before or after 12 am (OR = 0.063, 95% CI: 0.018–0.185) were all independent predictive factors for HM (Figure 8A). The logistic regression formula was


$$\begin{array}{l} Logistic\;\left(P\right)=\ln\left(\frac{P}{1-P}\right)=15.16-0.130\;vessel\\ densityofinnerretina\;at\;macula-\\ 0.271\;vesseldensityof\;RPC\;at\;\\ opticdisk+0.506\;smartphone\\ usagetime+0.793\;Continuous\\ nearworktime-2.766\;\\ sleepingafter\;12\;am. \end{array}$$

Based on these results, a prediction model for HM was established, with an AUC of 0.940 and 95% CI of 0.908–0.972 (*p* < 0.001, Figure 8B).

## 4. Discussion

The current study, for the first time, identified the influencing factors associated with HM in Chinese college students. Following the OCTA examination, the questionnaire survey, and the statistical analyses, the vessel density of the inner retina at the macula, the vessel density of RPC at the optic disc, the smartphone usage time, the continuous near work time, and sleeping after midnight have been determined as the influencing factors associated with HM for Chinese college students, including undergraduates and graduates in universities. Furthermore, a prediction model comprising these 5 influencing factors was proposed to calculate the likelihood of a college student to develop HM, according to which reasonable improvements in lifestyles and study habits could be recommended and the necessary medicinal and/or optometric interventions be implemented to decrease the incidence and slow the progression of HM.

Most studies on myopia have focused on the macula alone (23), while the current study examined the vessel density and structure thickness at both the macula and the optic disc of the same myopic eyes, whereby more comprehensive information could be obtained to predict the trend of myopia development and progression. This study found that the ensemble vessel density of the inner retina (from the ILM to 9 μm below the OPL) at the macula and the ensemble vessel density of the RPC at the optic disc were highly significantly reduced in the HM subjects in comparison to the non-HM subjects (Figures 7A,B). These results were consistent with a previous study by our research group, in which the decreased vessel density of the inner retina including retinal ganglion cell layer was detected by an ultrawide-field OCTA device in subjects with HM compared to normal controls (19), as well as consistent with other studies from different research groups (24, 25). Therefore, the diminished vessel density, indicative of insufficient blood perfusion (26), in the inner retina might be associated with progression and development of HM.

Indeed, the results of ROC analysis and univariant and multivariant logistic analyses confirmed that reduced ensemble vessel densities at both the macula and optic disc were influencing factors associated with HM in the Chinese college students (Figures 7, 8). On the other hand, although the retina thickness at the macula and the RPC thickness at the optic disc also exhibited significant decreases in the HM group compared to the non-HM group (Supplementary Figures 1A,B), their statistical significance was not as great as that of the vessel densities (Figures 7A,B). Their sensitivity and specificity as influencing factors for HM were also not as good, according to the ROC values (both <0.700, Supplementary Figures 3A,B). Therefore, the parameters of structure thickness at the macula and optic disc were excluded from further analyses of influencing factors.

However, it is of note that significant difference was found between non-HM and HM subjects in vessel density and retina thickness at peri- or parafovea regions, but not at the fovea (Figures 1–5). A possible explanation for the results of vessel density is that arterial blood supply is different in each part of the retina, and the fovea is an avascular zone with active local metabolism, thereby maintaining its blood flow in a relatively stable state (24). While the changes in structural thickness at different regions of the retina might be secondary to the changes in their corresponding blood supply, and vice versa, steady blood supply at fovea might result in unaltered retinal thickness at this part (27).

As for the parameters in lifestyle, the customized questionnaire survey results revealed that smartphone usage time was an influencing factor (Figure 7C), but computer usage time was not (Supplementary Figure 2B), suggesting that not all electronic devices contribute equally to the occurrence and progression of HM (28). Such differential effects could be due to the following reasons. First, although the radiation emitted from a smartphone is almost identical to that from a computer (29), the radioactive effects of smartphones on the eyes may be greater than those of computers as a result of the shorter distance entailed during smartphone usage. In fact, using an electronic device less than 20 cm in front of the eyes has been confirmed as an influencing factor for myopia progression and development (30). Therefore, using a smartphone is more deleterious to eye health than using a computer. Second, the reading posture during electronic device usage may also account for the differential effects. Specifically, when using a computer, people tend to look up (superior gaze) or look at the front horizontally (primary gaze); in contrast, people look down (downward gaze) in most cases of using a smartphone. Research has shown that the superior gaze elicits a slight decrease in the AL, whereas the downward gaze with the eye turn induces a significant elongation of the AL (31). Thus, the long-term usage of smartphones with downward gaze can promote myopia progression and HM exacerbation via AL elongation.

More interestingly, we identified that sleeping after midnight was an influencing factor for HM in Chinese college students, with more than 85% of the high myopes sleeping after midnight and more than two-thirds of the nonhigh myopes sleeping before midnight (Figure 7E). The possible mechanism underlying this observation could be ascribed to the 12-h diurnal rhythm of AL in adult humans, which reaches the peak at noon and touches the trough at midnight when the light is off (32, 33). In other words, under daily lighting conditions, the human AL is supposed to be the longest at noon and shortest at midnight. Therefore, it is speculated that sleeping after midnight could extend the light illumination, which might cause a phase delay of the diurnal rhythm and prevent AL diminution, eventually promoting AL elongation and myopia progression if sleeping after midnight becomes a lifestyle. However, this speculation awaits further investigation.

## 5. Limitations

There are some limitations in this study. This study is a small sample-sized, single-center, cross-sectional observational study. Large sample-sized, multicenter, and multiethnic group clinical trials are needed to validate the current results and the reliability of the prediction model. Furthermore, prospective cohort studies are also necessary to ascertain the pathogenic factors of HM in Chinese college students.

## 6. Conclusion

Taken together, the vessel density of the inner retina at the macula, the vessel density of RPC at the optic disc, smartphone usage time, continuous near work time, and sleeping after midnight have been identified as influencing factors associated with HM in Chinese college students. A prediction model constituted by these 5 influencing factors has been proposed to evaluate the odds of HM in Chinese college students.

## Data availability statement

The original contributions presented in the study are included in the article/Supplementary material, further inquiries can be directed to the corresponding authors.

## Ethics statement

The studies involving human participants were reviewed and approved by The Ethical Review Committee of Tianjin Medical University Eye Hospital (approval number 2022KY(L)-13). The patients/participants provided their written informed consent to participate in this study.

## Author contributions

ZL and YZ had full access to the data in the study, take responsibility for the integrity of the data and the accuracy of the data analysis, conceptualized and designed the research, and supervised the study. WZ, XH, CL, SW, and NL acquired and sorted the data. WZ wrote the draft of the manuscript. YZ critically reviewed, extensively revised, and submitted the manuscript. WZ and ZL performed the statistical analyses. YZ, NL, and ZL acquired fundings. All authors contributed to the article and approved the submitted version.

## Funding

This study was supported by the Tianjin Medical University Students’ Innovation and Entrepreneurship Training Program (TMUUROP2021-44), the project from National Natural Science Foundation of China (81970827), and the Tianjin Key Medical Discipline (Specialty) Construction Project (TJYXZDXK-037A).

## Conflict of interest

The authors declare that the research was conducted in the absence of any commercial or financial relationships that could be construed as a potential conflict of interest.

## Publisher’s note

All claims expressed in this article are solely those of the authors and do not necessarily represent those of their affiliated organizations, or those of the publisher, the editors and the reviewers. Any product that may be evaluated in this article, or claim that may be made by its manufacturer, is not guaranteed or endorsed by the publisher.
