# Supplementary material for: Influencing factors associated with high myopia in Chinese college students
Source: Front Med (Lausanne). 2023 Jun 23;10:1146291. doi: 10.3389/fmed.2023.1146291 (PMC10326280; doi:10.3389/fmed.2023.1146291)
Supplement: Supplementary file 1 [file Data_Sheet_1.pdf]

Supplementary figures

Supplementary figure 1

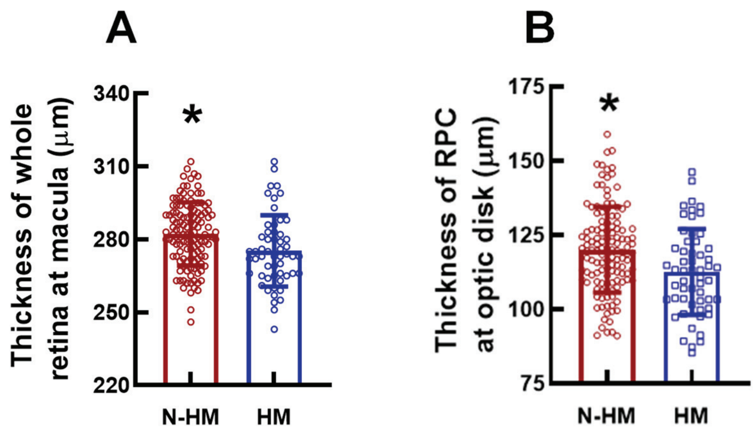

Supplementary figure 1. Comparison of structural thicknesses between non-HM and HM groups. The thicknesses of the whole retina at the macula were compared between the 2 groups **(A)**. The thicknesses of RPC at the optic disc were compared between the 2 groups **(B)**. * $p < 0.05$. non-HM, nonhigh myopia; HM, high myopia; RPC: radial peripapillary capillary.

Supplementary figure 2

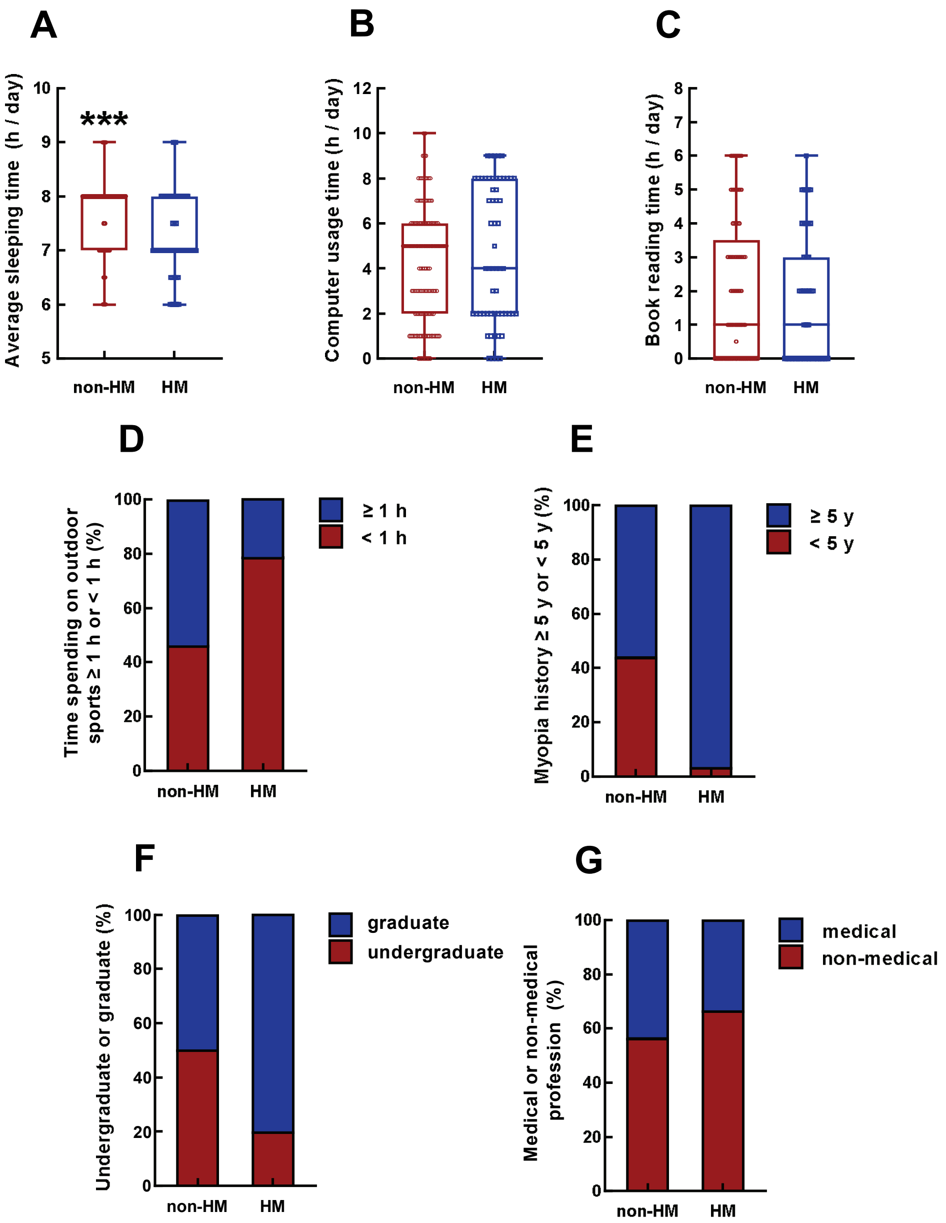

Supplementary figure 2. Comparison of the parameters in the questionnaire that had not been selected for further analysis. The average sleeping time **(A)**, computer usage time **(B)**, book reading time **(C)**, time spending on outdoor sports ⩾ or < 1 h **(D)**, history of myopia ⩾ or < 5 y **(E)**, undergraduate or graduate students **(F)**, and medical or nonmedical profession **(G)** were compared between the non-HM and HM groups. *** $p < 0.001$. non-HM, nonhigh myopia; HM, high myopia.

Supplementary figure 3

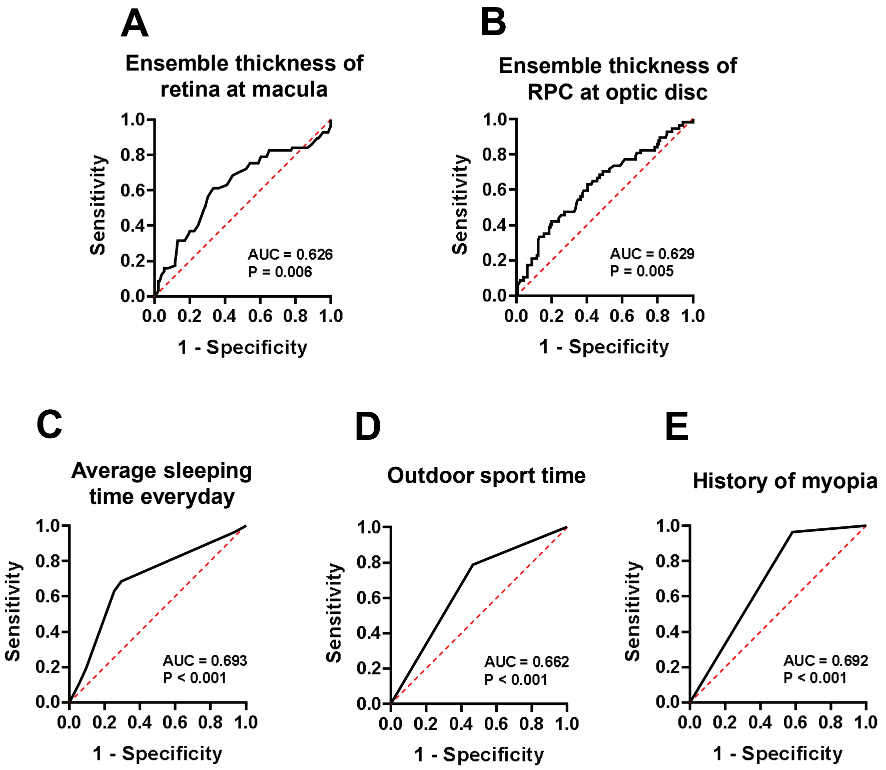

Supplementary figure 3. ROC curves of the factors with AUC less than 0.7. The ROC curves of retina thickness at the macula **(A)**, thickness of RPC at the optic disc **(B)**, average sleeping time **(C)**, outdoor sport time **(D)**, and history of myopia **(E)** are shown. ROC, receiver operator characteristic; AUC, area under the curve.
